# Supplementary material for: Efficient Screening of Organic Singlet Fission Molecules Using Graph Neural Networks
Source: Adv Sci (Weinh). 2026 Apr 28:e24389. Online ahead of print. doi: 10.1002/advs.202524389 (PMC13335501; doi:10.1002/advs.202524389)
Supplement: Supplementary file 1 — Supporting file: advs75469‐sup‐0001‐SuppMat.docx. [file ADVS-9999-e24389-s001.docx]

**Supporting Information**

**Efficient Screening of Organic Singlet Fission Molecules Using Graph Neural Networks**

Li Fu^1^, Longfei Lv^1^, Fan Zhang^2^, Si Zhou^1^, Weiwei Gao^3, 4*^, Jijun Zhao^1^^[[1]](#footnote-1)^

*^1^ Guangdong Basic Research Center of Excellence for Structure and Fundamental Interactions of Matter, Guangdong Provincial Key Laboratory of Quantum Engineering and Quantum Materials, School of Physics, South China Normal University, Guangzhou 510006, China*

*^2^ School of Physics, Nanjing University, Nanjing 210093, China*

*^3^ Institute of Atom Manufacturing, Nanjing University, Suzhou 215163, China*

*^4^ Nanjing Institute of Atomic Scale Manufacturing, Nanjing 211800, China*

**S1. Molecular geometric features analysis**

Molecular geometric features play a crucial role in determining molecular properties. To quantify the influence of geometric characteristics on excited-state properties, we use several descriptors that characterize molecular geometry, including the number of six-membered rings, the degree of molecular planarity, and quantitative measures of aromaticity.

The degree of molecular planarity is an important geometric characteristic of molecular structures and is closely related to aromaticity, electrical conductivity, and optical properties. Lu [1] proposed two simple metrics to evaluate molecular planarity⎯⎯the molecular planarity parameter (MPP) and the span of deviation from plane (SDP). Specifically, a best-fitting plane is first determined based on the atomic coordinates of the molecule by a least-squares procedure, yielding a plane that passes through the molecular geometric center and minimizes the distances to all atoms. This plane can be expressed as $Ax+By+Cz+D=0$ and the MPP is defined as the square root of the average of the squared distances of all atoms from this plane:

$$MPP=\sqrt{\frac{1}{N_{atom}}\sum_{i} d_{i}^{2}}$$

where $d_{i}$ denotes the distance of the *i-*th atom from the plane, which is given by:

$$d_{i}=\frac{\left| Ax_{i}+By_{i}+Cz_{i}+D \right|}{\sqrt{A^{2}+B^{2}+C^{2}}}$$

Considering that atoms may be located on either side of the fitted plane, the absolute value in the above expression is removed so that atoms on opposite sides of the plane can be distinguished by their signs. Accordingly, a signed distance$d_{i}^{s}$, which retains the sign information, is defined as:

$$d_{i}^{s}=\frac{Ax_{i}+By_{i}+Cz_{i}+D}{\sqrt{A^{2}+B^{2}+C^{2}}}$$

The span of deviation from plane (SDP) is obtained as the difference between the distances of the two atoms that are farthest from the fitted plane on opposite sides of the plane, as expressed by:

$$SDP=d_{max}^{s}-d_{min}^{s}$$

According to the definitions of MPP and SDP, both parameters are equal to zero for molecules in which all atoms lie in the same plane. In other words, for highly planar molecules, these two parameters are very small or approach zero. Larger values of MPP and SDP generally indicate greater deviations of atomic positions from the plane, corresponding to a more three-dimensional molecular geometry.

Molecular aromaticity plays a crucial role in understanding SF properties[2-4]. Assessing aromaticity in molecular systems is a highly complex task and can be approached using various methods, such as criteria based on Hückel’s rule [5], analyses of responses to external magnetic fields [6], and energy-based approaches [7]. However, these methods generally require quantitative calculations based on optimized molecular geometries and are therefore computationally expensive when applied to a large number of candidate molecular structures. In this work, we employ the harmonic oscillator measure of aromaticity (HOMA) evaluated on six-membered rings to quantify molecular aromaticity. This descriptor relies solely on geometric structural information and reflects aromatic character through variations in bond lengths, without the need for additional electronic-structure calculations [8]. Its expression is given by

$$HOMA=1-\sum_{i} \frac{\alpha_{i,j}}{N}(R_{ref}-R_{i,j})$$

Here, $R_{i,j}$ denotes the actual bond length between atoms and within a ring, and *N* is the number of atoms in the ring (in this work, only six-membered rings are considered, and thus $R_{ref}$represents the equilibrium bond length of atoms *i* and *j* in an ideal aromatic system, and $\alpha_{i,j}$is a parameter also derived from the corresponding ideal reference system. Both $R_{ref}$and $\alpha_{i,j}$are atom-dependent constants. In this study, the coefficients provided in pyAroma4 [9] are adopted for calculations. According to the definition, HOMA values closer to 1 indicate stronger aromaticity of the ring, values approaching 0 correspond to non-aromatic character, whereas negative values signify antiaromaticity. For molecules in the FORMED database that contain one or more six-membered rings, the maximum, minimum, and average HOMA values over all rings are used to characterize the overall molecular aromaticity.


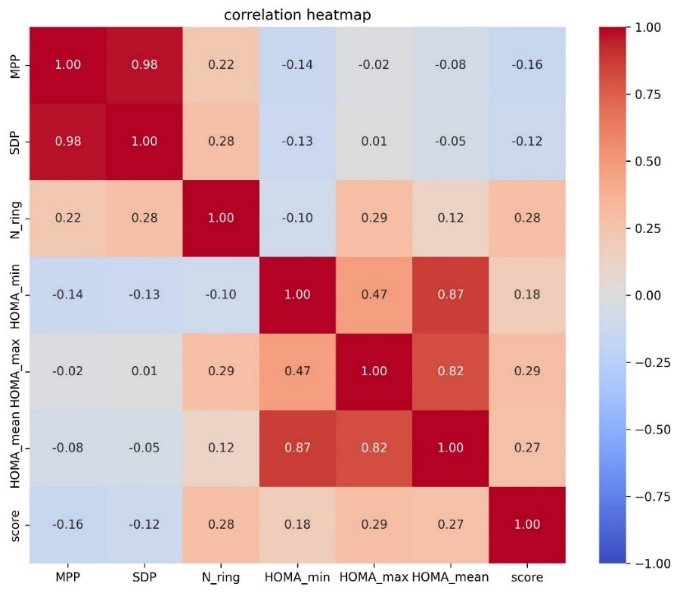


Figure S1. Correlation analysis between molecular planarity, aromaticity and their SF scores of molecules in the FORMED database. The planarity and aromaticity are quantified using molecular planarity parameter (MPP), span of deviation from plane (SDP), number of rings (N_ring), the minimum, maximum and mean values of harmonic oscillator measure of aromaticity (HOMA).

**S2. Details of graph neural network**


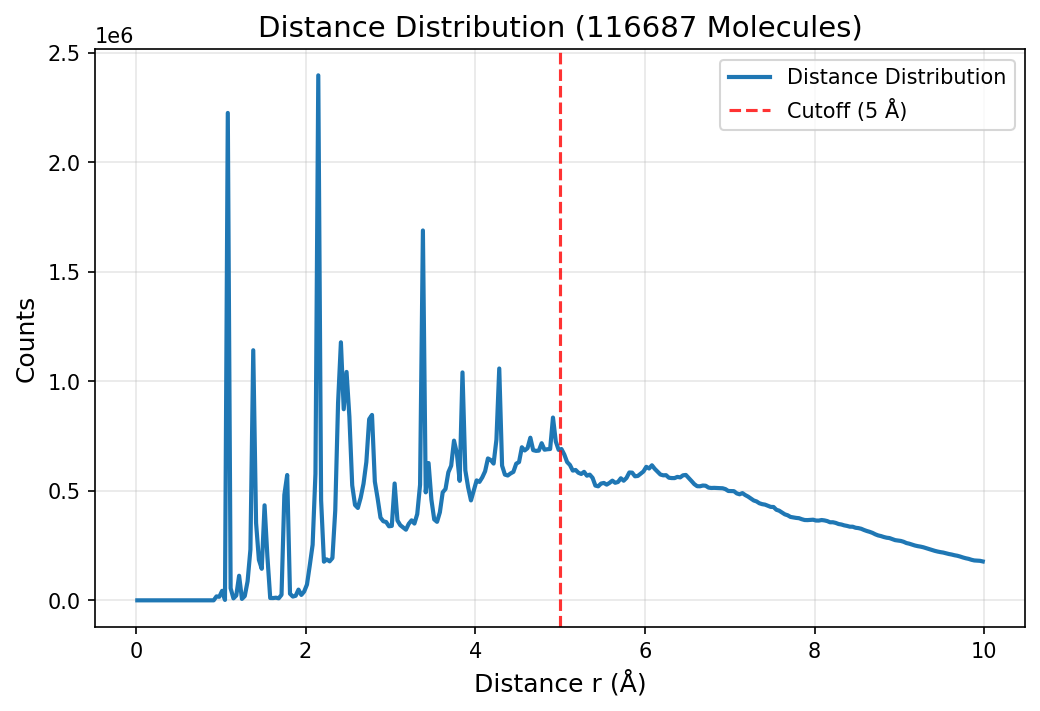


Figure S2. Interatomic distance distribution of 116687 molecules in the FORMED database.


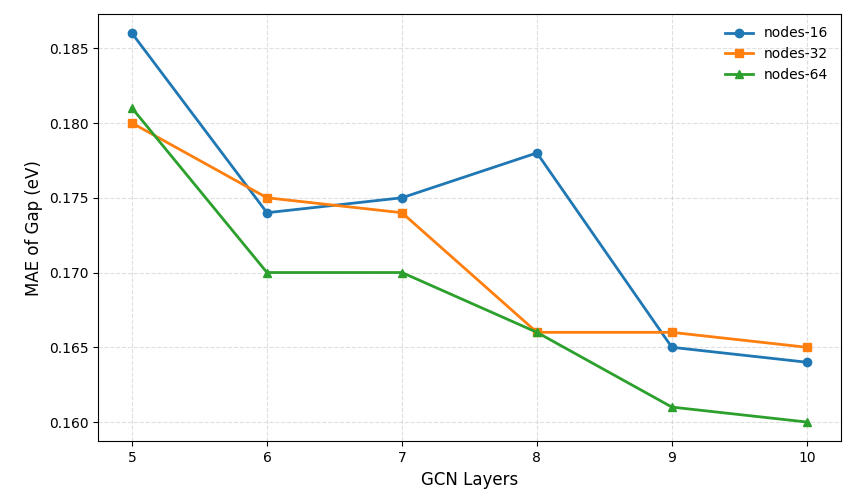


Figure S3. Hyperparameter optimization of GNN model in gap prediction task of FORMED datasets [10]. Mean absolute error (MAE) of gap as a function of the number of GCN layers and hidden layers.

**
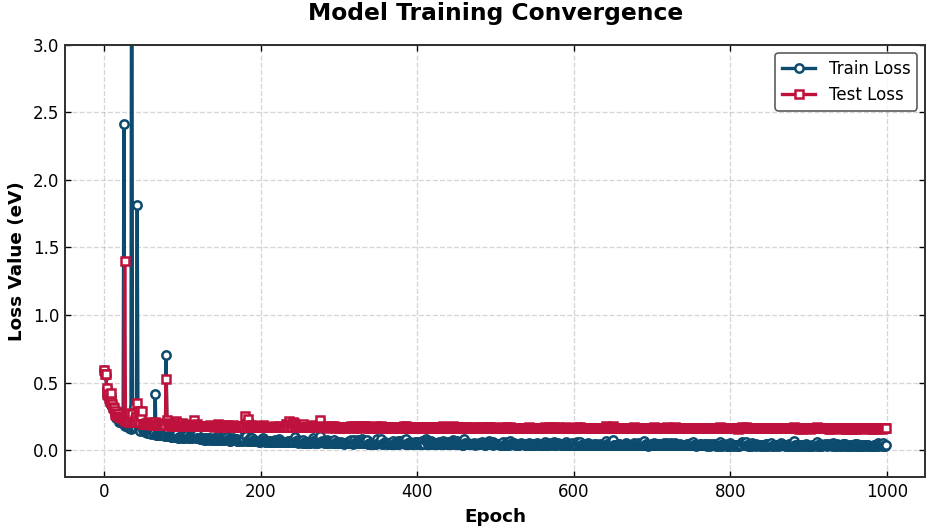
**

Figure S4. Mean absolute error (MAE, eV) of gap prediction model.


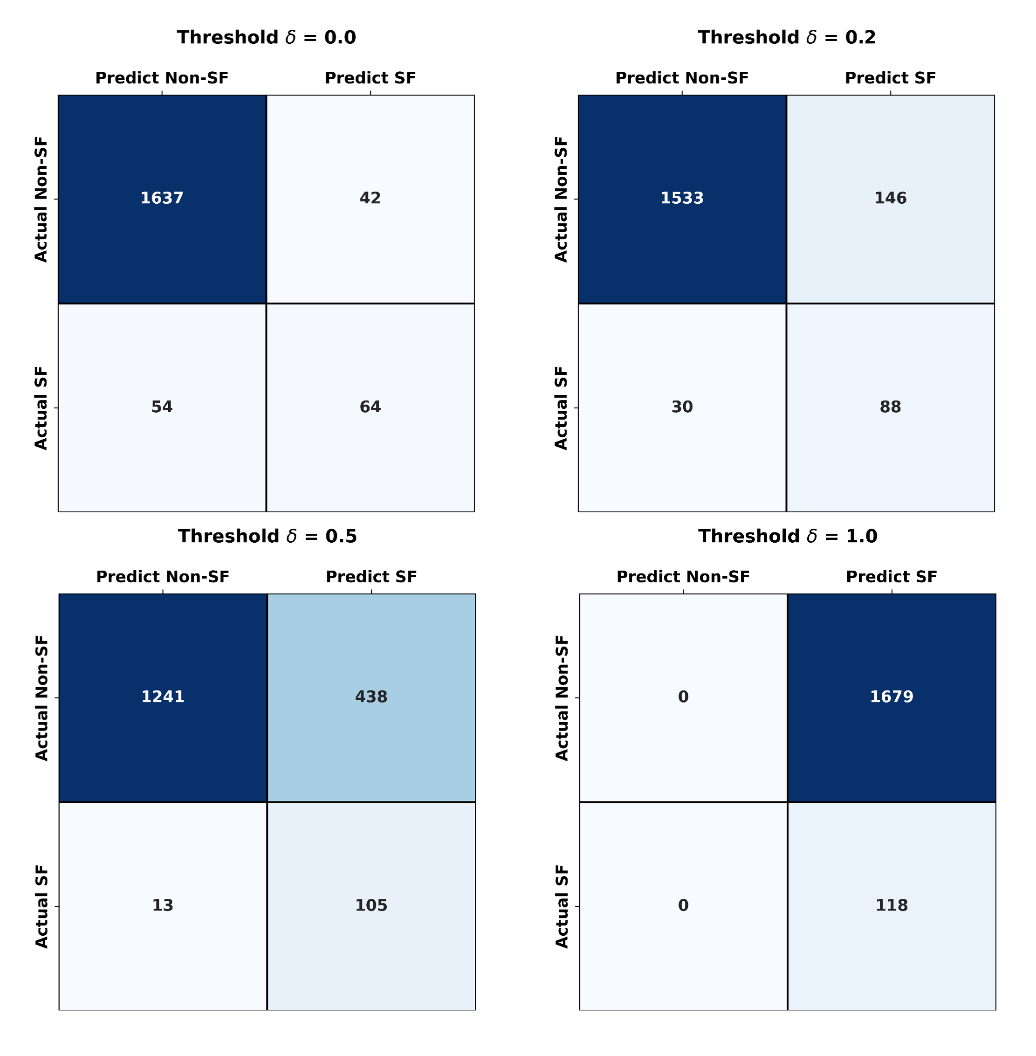


Figure S5. Confusion matrices for SF candidate screening at varying thresholds ($\boldsymbol{\delta}$ = 0, 0.2, 0.5, 1 eV).


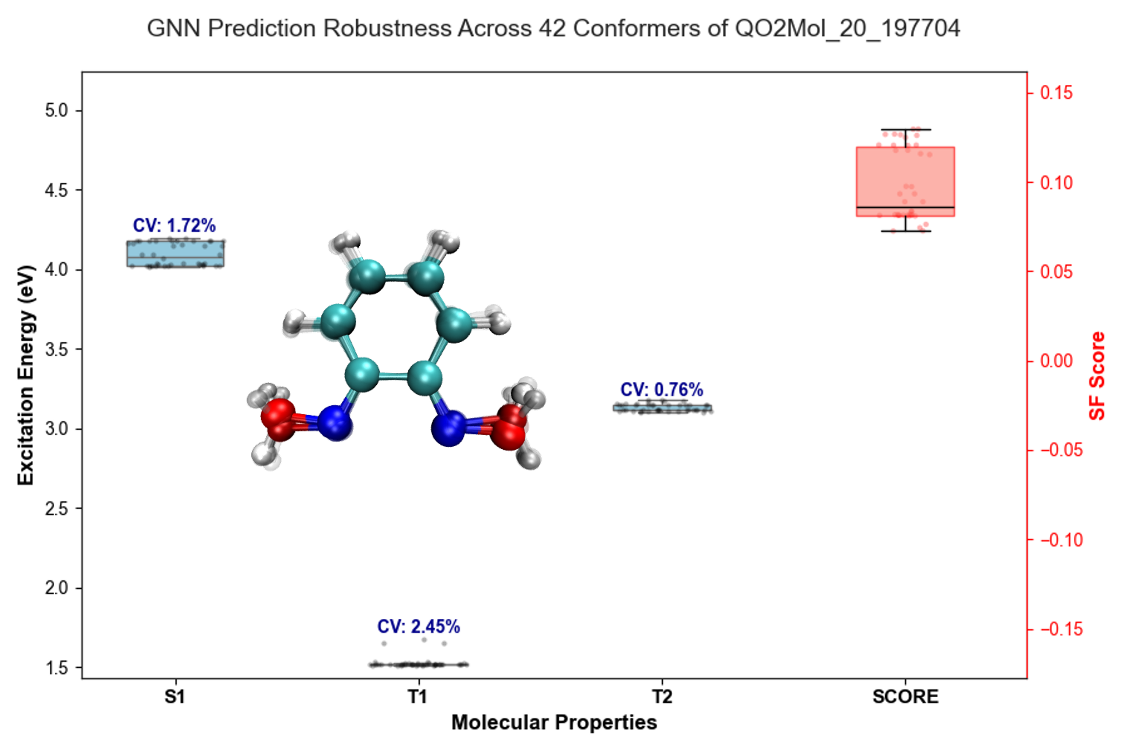


Figure S6. Assessment of GNN prediction stability across 42 distinct conformers of the molecule labeled as QO2Mol_20_197704. Boxplots and overlaid strip plots illustrate the minimal dispersion of predicted excitation energies (*S*_1_, *T*_1_, *T*_2_) and singlet fission score (red box). The corresponding coefficient of variation (CV) values, all below 2.50%, provide quantitative evidence that the model is remarkably robust against conformational distortions.


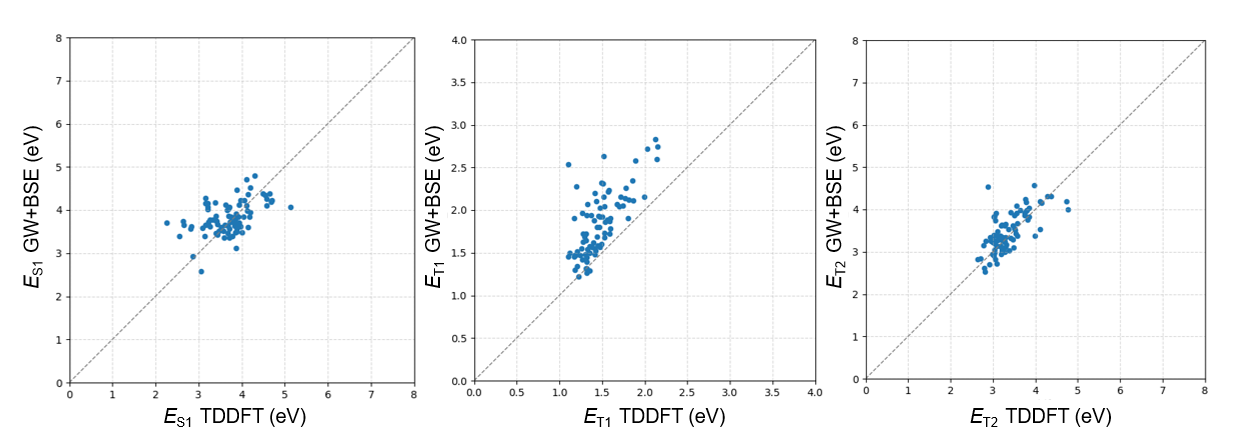


Figure S7. Comparison of excited-state energy levels of 79 easy synthesizable SF molecules calculated using TDDFT and GW+BSE method.

**S3. Structure and properties of molecules**


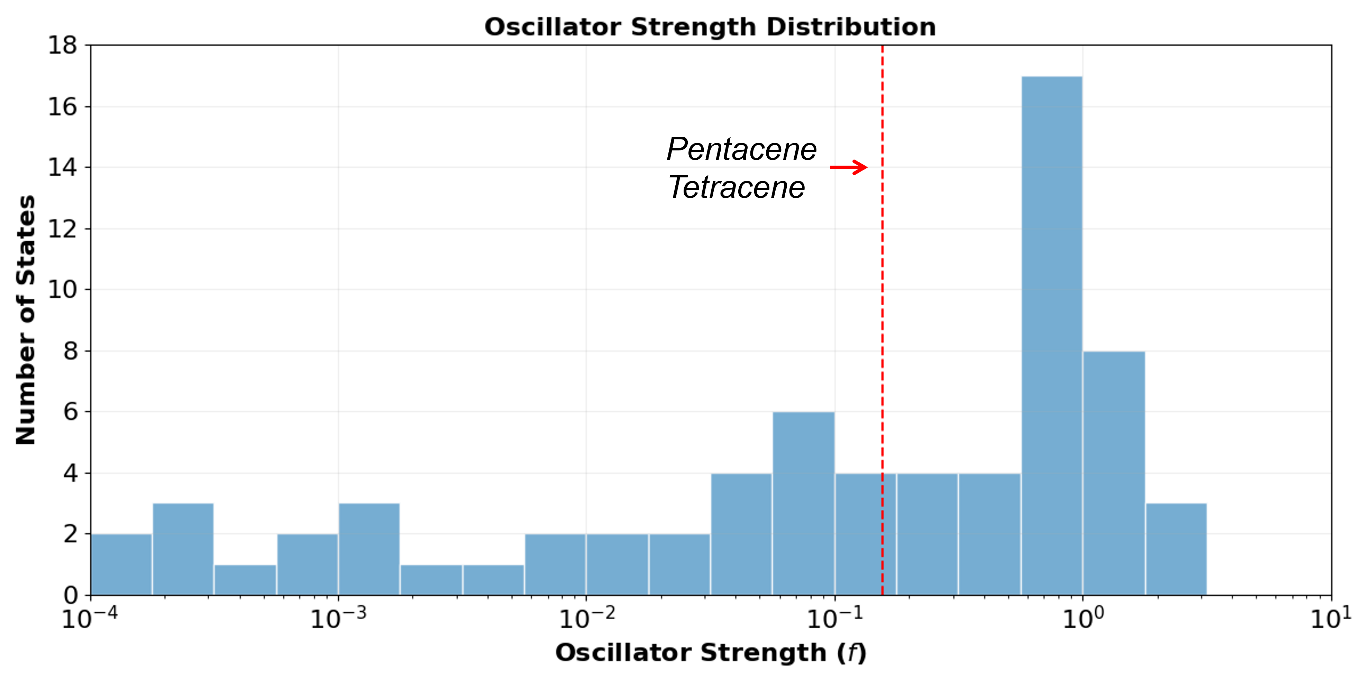


Figure S8. Distribution of *S*_1_ oscillator strength of 79 SF candidates (calculated at TDDFT level).


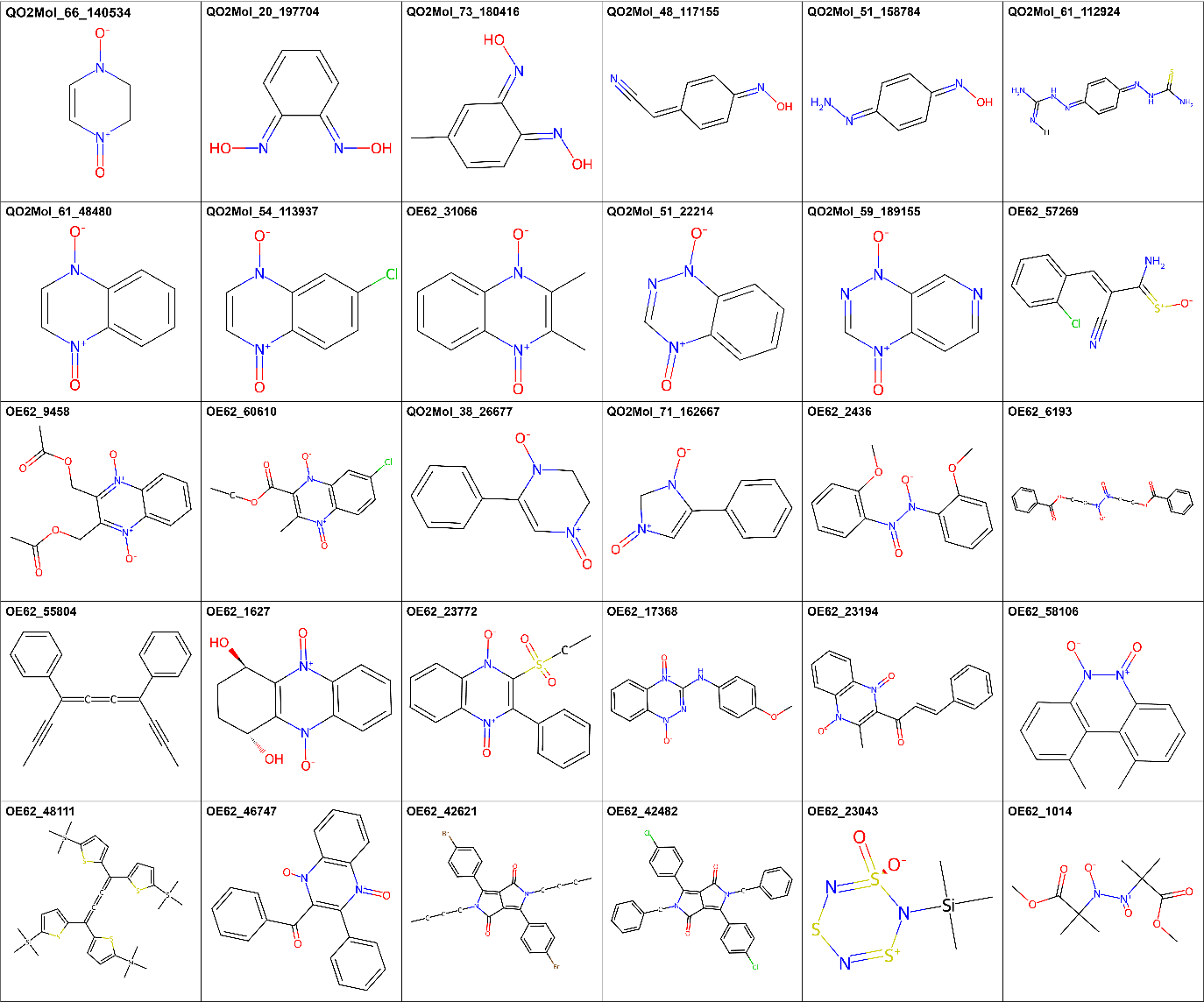


Figure S9. Planar diagram of 30 experimentally accessible SF molecules with positive SF scores from both TDDFT and GW+BSE calculations.

**S4.** $\boldsymbol{\delta}$ **thresholds tests**

Table S1. Performance of the SF screening protocol at various $\boldsymbol{\delta}$ thresholds, number of tasks for TDDFT calculations (*N*_TDDFT_) and number of true positives identified (SF mol) with recall and precision at different thresholds.

| $\boldsymbol{\delta}$ **(eV)** | ***N*_TDDFT_** | **SF mol** | **Recall** | **Precision** |
| --- | --- | --- | --- | --- |
| **0** | 107 | 64 | 54% | 60% |
| **0.2** | 237 | 88 | 75% | 38% |
| **0.5** | 553 | 105 | 89% | 19% |
| **1** | 1825 | 118 | 100% | 7% |

**S5. SF molecule and their conformers**

Table S2. SMILES of 118 SF candidates in OE62 database and 1080 SF candidates in QO2Mol database. The first column of the table lists SMILES strings, the second column presents the representative structures, and the third column gives all molecules sharing the corresponding SMILES string.

| **SMILES** | **Representative** | **All** |
| --- | --- | --- |
| [O]N(C(C(=O)OC)(C)C)N(C(C(=O)OC)(C)C)[O] | OE62_1014 | OE62_1014 |
| [O]N1N([C@@H]2CC[C@H]1C2)[O] | OE62_11827 | OE62_11827 |
| S1S[N]C(=C1C1=C(Cl)[N]SS1)Cl | OE62_13457 | OE62_13457 |
| c12c(cccc1)C(=O)[N]N2[N]c1ccc(cc1)N(CC)CC | OE62_13672 | OE62_13672 |
| c1(ccccc1)[C]1C(=O)O[N]S1 | OE62_13771 | OE62_13771 |
| S1CS[N]S[N]1 | OE62_13872 | OE62_13872 |
| N1=C2C=CC(=C[C]2N(S1)[O])N(=O)=O | OE62_15435 | OE62_15435 |
| C1C[C@@H](O)c2c([C@@H]1O)[n+](c1c(cccc1)[n+]2[O-])[O-] | OE62_1627 | OE62_1627 |
| c1cc([As]2[N]S[N]S2)ccc1 | OE62_16342 | OE62_16342 |
| Cl[C@H]1[C@@H]2C=C[C@H]([C@H]1N([O])N([O])[C@@H]1[C@@H]([C@H]3C=C[C@@H]1C3)Cl)C2 | OE62_1672 | OE62_1672 |
| C1C[C@H]2CC[C@@]1(C2)N(N([O])[C@@]12CC[C@@H](CC1)C2)[O] | OE62_16816 | OE62_16816 |
| C1C[C@@H]([C@@](C1)(Cl)[Si](C)(C)C)N([O])N([C@@H]1CCC[C@@]1(Cl)[Si](C)(C)C)[O] | OE62_16959 | OE62_16959 |
| [O]N([C@H]1[C@@H]2CC[C@@H](C2)[C@]1(Cl)[Si](C)(C)C)N([O])[C@@H]1[C@H]2CC[C@H](C2)[C@@]1(Cl)[Si](C)(C)C | OE62_16962 | OE62_16962 |
| [O-][n+]1c2ccccc2[n+]([O-])nc1Nc1ccc(OC)cc1 | OE62_17368 | OE62_17368 |
| c1c2C3=CC=C[CH]C3=C(c2c(cc1)Cl)C1=c2c(Cl)cccc2=C2C1=CC=C[CH]2 | OE62_17510 | OE62_17510 |
| n1c2c(cccc2C)[n+]([O-])c2c1c(ccc2)C(C)(C)C | OE62_18562 | OE62_18562 |
| ClC1=CC=C(C2=NON([O])[C]12)Cl | OE62_18939 | OE62_18939 |
| CC(N([O])N(C(C(=[NH2])[N]C#N)(C)C)[O])(C(=[NH2])[N]C#N)C | OE62_19742 | OE62_19742 |
| O=C1O[N]N([C]1C1=CCCCC1)c1ccccc1 | OE62_20845 | OE62_20845 |
| [C]1(c2ccccc2)SC(=C2[N]N(C)N=C12)c1ccccc1 | OE62_21187 | OE62_21187 |
| N#C[C](C#N)C1=NS[N]S1 | OE62_21559 | OE62_21559 |
| c1cccc2c1C(=O)/C(=C/1\Nc3c(C1=O)cccc3)/N2 | OE62_2161 | OE62_2161 |
| c1ccccc1NC1=CC(=C2C(=O)c3ccccc3C(=C2C1=O)Nc1ccccc1)O | OE62_21616 | OE62_21616 |
| N(=[S][N]Sc1cc(Cl)ccc1)Sc1cc(Cl)ccc1 | OE62_22523 | OE62_22523 |
| [O]N1[C](c2ccccc2)C(=O)c2ccc(OC)cc12 | OE62_22898 | OE62_22898 |
| S1[N]S[N]S(=O)(=O)N1[Si](C)(C)C | OE62_23043 | OE62_23043 |
| [O-][n+]1c2ccccc2[n+](c(c1C(=O)/C=C/c1ccccc1)C)[O-] | OE62_23194 | OE62_23194 |
| O=C1c2c(C(=O)c3ccccc13)c(NCC)ccc2NCC | OE62_23433 | OE62_23433 |
| [O-][n+]1c2c([n+]([O-])c(S(=O)(=O)CC)c1c1ccccc1)cccc2 | OE62_23772 | OE62_23772 |
| [O]N(N([O])c1ccccc1OC)c1ccccc1OC | OE62_2436 | OE62_2436 |
| O=N(=O)[C]1C=C(N(=O)=O)C=C2N(ON=C12)[O] | OE62_24819 | OE62_24819 |
| S([N]S[N]c1ccc(OC)cc1)c1ccc(N(=O)=O)cc1 | OE62_25261 | OE62_25261 |
| Clc1cc(C2=C3C(=O)NC(=C3C(=O)N2)c2cccc(Cl)c2)ccc1 | OE62_25704 | OE62_25704 |
| S([N]Sc1ccccc1)[N]c1ccccc1 | OE62_26595 | OE62_26595 |
| O(C(=O)C1=C[C](C(=O)OC)[C@]2([C@@]1(C(=C[C]2C(=O)OC)C(=O)OC)C)C)C | OE62_28730 | OE62_28730 |
| C1c2c(C)ccc(c2C(=O)[C](C1(C)C)S[N]C(C)(C)C)C | OE62_28958 | OE62_28958 |
| c1coc(CN2N([O])[C]3C(=C2C#N)C=CC=C3)c1 | OE62_29935 | OE62_29935 |
| N#C[C](C#N)c1cc2O[C]3C(=C(c2cc1)c1c(C)cc(cc1C)OC)C=CC=C3 | OE62_30356 | OE62_30356 |
| C1(=O)C2=C(N(CC=C)C(=O)C2=C(N1CC=C)c1ccccc1)c1ccccc1 | OE62_30386 | OE62_30386 |
| [O-][n+]1c2ccccc2[n+]([O-])c(c1C)C | OE62_31066 | OE62_31066 |
| C12=CC(=C[C](C2=NON1[O])N(=O)=O)C#N | OE62_31610 | OE62_31610 |
| [Se]1[C](C=C(C=C1c1ccccc1)C1=C[C](c2ccccc2)[Se]C(=C1)c1ccccc1)c1ccccc1 | OE62_31714 | OE62_31714 |
| c1cccc(C/C=C/C(=C(/C=C/Cc2ccccc2)\C#CC(C)(C)C)/C#CC(C)(C)C)c1 | OE62_31920 | OE62_31920 |
| FC1=C(F)C=C(F)[C]2C1=NS[N][Se]2 | OE62_32132 | OE62_32132 |
| c1ccc2c3[N+]([O-])C(=O)N(C)[N]c3ccc2c1 | OE62_34021 | OE62_34021 |
| C1(=O)C2=C(c3ccccc3)N(C(=O)C2=C(N1C)c1ccccc1)c1ccc(cc1)OC | OE62_34322 | OE62_34322 |
| C(C)(N(N([O])C(C)(C)C)[O])(C)C | OE62_345 | OE62_345 |
| N#C/C(=C\C1=C2[CH]C=CC=C2N(C=C1)C)/[C](C#N)C#N | OE62_34744 | OE62_34744 |
| C\1(=N/O)/C=CC=C/C1=N/O | OE62_34944 | OE62_34944 |
| Cc1ccc(c2c3CCCCCCc(c12)c1c(ccc(c31)C)C)C | OE62_35028 | OE62_35028 |
| ClCCCN1C(=O)C2=C(N(CCCCl)C(=O)C2=C1c1c(OC)cccc1)c1c(OC)cccc1 | OE62_36154 | OE62_36154 |
| O[C]1C(=O)Oc2c1cc1OC(=O)[C](c1c2)c1ccccc1 | OE62_37197 | OE62_37197 |
| c1([N]C2=NC(=C[C]2c2ccccc2)c2ccccc2)[nH]c(cc1c1ccccc1)c1ccccc1 | OE62_37690 | OE62_37690 |
| O=S(=O)([N]S[C](Sc1ccccc1)c1c(cc(cc1C)C)C)c1ccc(cc1)C | OE62_38058 | OE62_38058 |
| N/C(=C\[C]1C(=O)[C](C1=O)c1ccc(cc1)N(CCCC)CCCC)/C | OE62_38303 | OE62_38303 |
| [C@H]12[C@@H]3[C@@H]4C[C@H]1[C@H](N([O])N2[O])[C@H]34 | OE62_38744 | OE62_38744 |
| O1N(C2=CC=C[CH]C2=N1)[O] | OE62_38973 | OE62_38973 |
| C1(=O)CCCC[C@@]1(C(=O)OCC)N([O])N([C@@]1(C(=O)CCCC1)C(=O)OCC)[O] | OE62_39434 | OE62_39434 |
| Fc1c(F)c(F)c(F)c(c1F)[C](c1c2c(c(C(=O)OCC)[nH]1)cccc2)C1=NC(=C2[C]1C=CC=C2)C(=O)OCC | OE62_39543 | OE62_39543 |
| [O]N(N([C@H]1[C@@H]([C@H]2C[C@@H](C1)C2(C)C)C)[O])[C@H]1[C@@H]([C@H]2C[C@@H](C1)C2(C)C)C | OE62_40302 | OE62_40302 |
| N(/C=C/[CH]c1c(O)cccc1)(c1ccc(C)cc1)[O] | OE62_40747 | OE62_40747 |
| C[Si](/[Si](=C\1/[C@@H]2C[C@@H]3C[C@H]1C[C@H](C2)C3)/[Si](C)(C)C)(C)C(C)(C)C | OE62_40761 | OE62_40761 |
| c12c(c3ccccc3c(c2c(c2c(c1Cl)cccc2)Cl)Cl)Cl | OE62_4173 | OE62_4173 |
| O(C(=O)c1c2[C](C#N)S[C](c2c(s1)C(=O)OC)C#N)C | OE62_41772 | OE62_41772 |
| C12=CC(O[N@@+]([O-])[C]2CCCC1)(C)C | OE62_41877 | OE62_41877 |
| c1cccc(CN2C(=C3C(=O)N(C(=C3C2=O)c2ccc(Cl)cc2)Cc2ccccc2)c2ccc(Cl)cc2)c1 | OE62_42482 | OE62_42482 |
| Brc1ccc(cc1)C1=C2C(=C(N(C2=O)CCCC)c2ccc(Br)cc2)C(=O)N1CCCC | OE62_42621 | OE62_42621 |
| N1(C(=O)C2=C(c3ccc(cc3)C#C)N(C(=O)C2=C1c1ccc(cc1)C#C)CCCC)CCCC | OE62_42623 | OE62_42623 |
| C\1(=C/[CH]C2=C[C](C(C)(C)C)OC(=C2)C(C)(C)C)/C(=O)ON=C1c1ccccc1 | OE62_43139 | OE62_43139 |
| [O]N(N([C@@]12[C@@]3(N(=O)=O)C[C@@H](C1)C[C@H](C3)C2)[O])[C@]12C[C@@H]3C[C@@]1(N(=O)=O)C[C@H](C2)C3 | OE62_43791 | OE62_43791 |
| CCCN(CCC)c1cc(c(cc1)[C]1C(=O)[C](c2ccc(cc2O)N(CCC)CCC)C1=O)O | OE62_45594 | OE62_45594 |
| c1cccc([C@@H]2N([N]c3c([C]2c2ccccc2)cc(Cl)cc3/C(=C\c2ccccc2)/c2ccccc2)c2ccc(cc2)Cl)c1 | OE62_46183 | OE62_46183 |
| C(#C[C](C#C[Si](C(C)C)(C(C)C)C(C)C)c1ccccc1)[C](c1ccccc1)C#C[Si](C(C)C)(C(C)C)C(C)C | OE62_46624 | OE62_46624 |
| O=C(c1c(c2ccccc2)[n+]([O-])c2c([n+]1[O-])cccc2)c1ccccc1 | OE62_46747 | OE62_46747 |
| [C@@H]1(CCCC[C@@H]1N(N([O])[C@@H]1[C@H](CCCC1)N(=O)=O)[O])N(=O)=O | OE62_4690 | OE62_4690 |
| c1c(N(=O)=O)ccc2[N]C3(CCCCC3)N([O])c12 | OE62_471 | OE62_471 |
| C1=C[C@H](c2ccccc2)/C(=C/c2ccccc2)/[C]([N+]1[O-])c1ccccc1 | OE62_48012 | OE62_48012 |
| s1c(ccc1[C](C#C[C](c1sc(cc1)[Si](C)(C)C)c1sc([Si](C)(C)C)cc1)c1sc([Si](C)(C)C)cc1)[Si](C)(C)C | OE62_48111 | OE62_48111 |
| N1=C[C]([S]=O)C2(N1)C(C)(CCCC2(C)C)C | OE62_48417 | OE62_48417 |
| c1c(C(C)(C)C)c([P]c2sc(cc2)[C](c2ccc(cc2)C)c2ccc(cc2)C)c(cc1C(C)(C)C)C(C)(C)C | OE62_48467 | OE62_48467 |
| n1ccnc2c1c(ccc2[C](C#N)C#N)[C](C#N)C#N | OE62_4847 | OE62_4847 |
| c1ccccc1C1=C2C(=C(N(C2=O)CCCCCC)c2ccccc2)C(=O)N1CCCCCC | OE62_48647 | OE62_48647 |
| BrC#C/C(=C(\C#C[Si](C(C)C)(C(C)C)C(C)C)/C#CBr)/C#C[Si](C(C)C)(C(C)C)C(C)C | OE62_48660 | OE62_48660 |
| [C]1([C@@H](N([N]c2c1cccc2/C(=C\c1ccccc1)/c1ccccc1)c1ccccc1)c1ccccc1)c1ccccc1 | OE62_48887 | OE62_48887 |
| c1ccc(cc1)c1c2c3CCCCCCc(c4c(ccc(c5ccccc5)c34)c3ccccc3)c2c(cc1)c1ccccc1 | OE62_49487 | OE62_49487 |
| C(C)CCN1C(=C2C(=O)N(C(=C2C1=O)c1ccccc1)CCCC)c1ccc(cc1)c1ccc(cc1)OC | OE62_50355 | OE62_50355 |
| O(c1cc2CCN=C(c2cc1OC)c1c(N([O])N(c2c(C3=NCCc4cc(OC)c(OC)cc34)cccc2)[O])cccc1)C | OE62_50629 | OE62_50629 |
| [Si](=[Si]([Si](C)(C(C)C)C(C)C)[Si](C)(C(C)C)C(C)C)([Si](C)(C)C(C)(C)C)[Si](C)(C)C(C)(C)C | OE62_51473 | OE62_51473 |
| CCCCCCCN(c1cc(O)c(cc1)[C]1C(=O)[C](c2ccc(N(CCCCCCC)CCCCCCC)cc2O)C1=O)CCCCCCC | OE62_51529 | OE62_51529 |
| N#C[C](c1ccc([C]2[N@]3CCC[C@H]3CN2)cc1)C#N | OE62_51738 | OE62_51738 |
| [Si]([Si](C)[Si](C(C)(C)C)(C(C)(C)C)C)([Si]([Si](C)(C(C)(C)C)C(C)(C)C)([Si](C)(C(C)(C)C)C(C)(C)C)C)[Si](C)(C(C)(C)C)C(C)(C)C | OE62_52530 | OE62_52530 |
| [Si](C#C/C(=C(\C#CC(=O)OCC(C)C)/C#C[Si](C(C)C)(C(C)C)C(C)C)/C#CC(=O)OCC(C)C)(C(C)C)(C(C)C)C(C)C | OE62_52651 | OE62_52651 |
| [Si](C(c1c(c(ccc1)C([Si](C)(C)C)[Si](C)(C)C)[SiH][SiH]c1c(C([Si](C)(C)C)[Si](C)(C)C)cccc1C([Si](C)(C)C)[Si](C)(C)C)[Si](C)(C)C)(C)(C)C | OE62_53393 | OE62_53393 |
| CC(c1cc(C(C)(C)C)cc(c1[P]/C(=C(/[P]c1c(cc(C(C)(C)C)cc1C(C)(C)C)C(C)(C)C)\C)/C)C(C)(C)C)(C)C | OE62_53463 | OE62_53463 |
| C[C]1c2ccccc2CN1[C](C#N)C#N | OE62_53890 | OE62_53890 |
| S([N]Sc1ccc(cc1)N(=O)=O)[N]c1c(c(c(Br)c(c1F)F)F)F | OE62_53903 | OE62_53903 |
| CC1=CC(C)(N(N1[O])[O])C | OE62_54389 | OE62_54389 |
| N#C[N]N([N]C#N)c1ccccc1 | OE62_5505 | OE62_5505 |
| COc1ccc(C2S[N]S[N]S2)cc1 | OE62_55160 | OE62_55160 |
| Brc1oc(C(=C2OC(=O)C(=C2)Br)c2ccc(Br)o2)cc1 | OE62_55448 | OE62_55448 |
| c1(ccccc1)[C](C#C[C](C#CC)c1ccccc1)C#CC | OE62_55804 | OE62_55804 |
| O=[S]/C(=C(/[CH]c1c(Cl)cccc1)\C#N)/N | OE62_57269 | OE62_57269 |
| c1cc(C)c2c(c1)[n+]([O-])[n+]([O-])c1c2c(ccc1)C | OE62_58106 | OE62_58106 |
| O=C1/C(=C\2/C(=O)c3ccc(Br)cc3N2)/Nc2cc(Br)ccc12 | OE62_59527 | OE62_59527 |
| c1cc(/C(=C\2/C(=N/O)/SC(=C2)c2ccccc2)/C#N)ccc1 | OE62_60116 | OE62_60116 |
| c1ccc([P]2([N]S[N]S[N]2)c2ccccc2)cc1 | OE62_60565 | OE62_60565 |
| [O-][n+]1c(c([n+]([O-])c2c1ccc(c2)Cl)C(=O)OCC)C | OE62_60610 | OE62_60610 |
| O=C(c1ccccc1)OCCN([O])N(CCOC(=O)c1ccccc1)[O] | OE62_6193 | OE62_6193 |
| c1cccc(N2[N]OC(=O)[C]2C(=C)C(C)C)c1 | OE62_7088 | OE62_7088 |
| CO[C@H]1[C@H]2[C@H]([C@]3(N(N([C@]2(C)C3(C)C)[O])[O])C)[C@H](O1)OC | OE62_7111 | OE62_7111 |
| N1(C2=C(C=C[C](C2=NO1)Br)Br)[O] | OE62_7546 | OE62_7546 |
| c1ccc(cc1)[N]S[N]c1c(c(c(F)c(c1F)F)F)F | OE62_8326 | OE62_8326 |
| O=C(OC)[C](c1ccc(cc1)[C](C(=O)OC)C(=O)OC)C(=O)OC | OE62_8345 | OE62_8345 |
| c1(c2c(cccc2)c(c2c1c(C)c1c(c2C)cccc1)C)C | OE62_9196 | OE62_9196 |
| C(=O)(C)OCc1[n+]([O-])c2ccccc2[n+](c1COC(=O)C)[O-] | OE62_9458 | OE62_9458 |
| Cl[C@@H]1[C@@H]([C@H]2CC[C@@H]1C2)N([O])N([O])[C@@H]1[C@H](Cl)[C@H]2CC[C@@H]1C2 | OE62_9501 | OE62_9501 |
| FC(F)(F)[C@H]1C[C@H]2[C@H](Cl)[C@@H]([C@@H]1C2)N(N([O])[C@@H]1[C@H](Cl)[C@H]2C[C@H]([C@@H]1C2)C(F)(F)F)[O] | OE62_9503 | OE62_9503 |
| C=C1C=C/C(=N/O)/C=C1 | QO2Mol_12_153647 | QO2Mol_12_153647,QO2Mol_18_152344,QO2Mol_23_177841,QO2Mol_83_201110,QO2Mol_22_32077 |
| [O]N1[CH]Sc2ccccc12 | QO2Mol_13_118083 | QO2Mol_13_118083 |
| [O]N1C=CN([O])C21CCCC2 | QO2Mol_13_98452 | QO2Mol_13_98452,QO2Mol_30_140393,QO2Mol_2_178020,QO2Mol_3_1855,QO2Mol_25_22102,QO2Mol_17_200888,QO2Mol_11_166730,QO2Mol_3_83517,QO2Mol_3_28930 |
| CN([O])[CH]c1cnc[nH]1 | QO2Mol_14_125532 | QO2Mol_14_125532 |
| N[N]c1nc(Cl)c([CH]c2ccco2)[nH]1 | QO2Mol_15_100540 | QO2Mol_15_100540,QO2Mol_24_90923,QO2Mol_14_201948,QO2Mol_26_44449,QO2Mol_3_126080,QO2Mol_13_158448,QO2Mol_30_123229,QO2Mol_28_191751,QO2Mol_23_113108,QO2Mol_9_664,QO2Mol_9_49928,QO2Mol_9_58513,QO2Mol_16_125154,QO2Mol_3_97110,QO2Mol_17_62562,QO2Mol_64_5008,QO2Mol_27_84906,QO2Mol_18_178483,QO2Mol_25_142817,QO2Mol_26_33894,QO2Mol_16_199344,QO2Mol_21_44369,QO2Mol_3_107487,QO2Mol_18_185889,QO2Mol_22_81092,QO2Mol_21_164640,QO2Mol_10_135819,QO2Mol_15_200100,QO2Mol_27_29645,QO2Mol_29_51685,QO2Mol_12_54384,QO2Mol_79_152111,QO2Mol_30_53916,QO2Mol_21_77886,QO2Mol_9_175987,QO2Mol_3_2891,QO2Mol_10_82448,QO2Mol_26_182736,QO2Mol_21_196973,QO2Mol_13_80428,QO2Mol_20_120877,QO2Mol_14_60480,QO2Mol_18_90535,QO2Mol_26_60401,QO2Mol_76_65545,QO2Mol_26_135008,QO2Mol_17_184226,QO2Mol_8_96711,QO2Mol_13_58978,QO2Mol_25_121785,QO2Mol_16_70997,QO2Mol_4_18413,QO2Mol_11_95184,QO2Mol_22_57635,QO2Mol_2_177631,QO2Mol_21_146900,QO2Mol_9_25558,QO2Mol_18_1582,QO2Mol_18_5759,QO2Mol_62_24964,QO2Mol_10_126893,QO2Mol_15_42892,QO2Mol_26_158162,QO2Mol_15_62571,QO2Mol_20_174754,QO2Mol_18_28109,QO2Mol_15_163225,QO2Mol_68_165338,QO2Mol_18_183362,QO2Mol_15_120558,QO2Mol_28_51859,QO2Mol_9_157595,QO2Mol_15_40303,QO2Mol_15_157384,QO2Mol_22_187305,QO2Mol_14_148764,QO2Mol_12_118666,QO2Mol_10_5484,QO2Mol_10_83569,QO2Mol_11_50727,QO2Mol_21_147313,QO2Mol_88_185363,QO2Mol_22_44773,QO2Mol_8_98269,QO2Mol_30_95488,QO2Mol_3_114307,QO2Mol_13_148501,QO2Mol_12_193240,QO2Mol_25_16897,QO2Mol_12_181357,QO2Mol_24_175951,QO2Mol_17_74965,QO2Mol_23_45192,QO2Mol_16_6968,QO2Mol_27_164312,QO2Mol_28_70770,QO2Mol_27_52754,QO2Mol_23_26414,QO2Mol_10_177358,QO2Mol_13_24106,QO2Mol_29_139604,QO2Mol_19_163346,QO2Mol_20_9995,QO2Mol_23_92753,QO2Mol_29_35899,QO2Mol_26_199218,QO2Mol_70_99864,QO2Mol_14_113646,QO2Mol_27_89536,QO2Mol_3_90380,QO2Mol_19_54162,QO2Mol_8_89180,QO2Mol_9_154244,QO2Mol_23_35140 |
| [O]N1[C](/C=C/c2ccccc2)CCC1 | QO2Mol_15_184828 | QO2Mol_15_184828,QO2Mol_3_67952 |
| O/N=C\1/C=C/C(=C/c2ccccc2)/C=C1 | QO2Mol_19_189718 | QO2Mol_19_189718,QO2Mol_22_3789,QO2Mol_30_19364,QO2Mol_21_119558,QO2Mol_17_67950,QO2Mol_8_74080,QO2Mol_21_54299,QO2Mol_19_199199,QO2Mol_63_140569,QO2Mol_16_11224,QO2Mol_20_93361,QO2Mol_21_119127,QO2Mol_19_75633,QO2Mol_9_37189,QO2Mol_8_173774,QO2Mol_15_40317,QO2Mol_23_30001,QO2Mol_15_110191,QO2Mol_88_185237,QO2Mol_10_202379,QO2Mol_11_3198,QO2Mol_23_116146,QO2Mol_17_48679,QO2Mol_28_200899,QO2Mol_11_197373,QO2Mol_3_86425,QO2Mol_30_116177,QO2Mol_29_179041,QO2Mol_2_134182,QO2Mol_8_191176,QO2Mol_19_81531,QO2Mol_17_49274,QO2Mol_12_180003,QO2Mol_30_150977,QO2Mol_20_91246,QO2Mol_24_107114 |
| [O]N(/C=C/1\N=C[N]S1)C1CCCCC1 | QO2Mol_19_91232 | QO2Mol_19_91232,QO2Mol_11_17935,QO2Mol_13_69966 |
| O/N=C/1\C=CC=C\C1=N/O | QO2Mol_20_197704 | QO2Mol_20_197704,QO2Mol_13_1724,QO2Mol_19_14682,QO2Mol_24_10907,QO2Mol_9_134262,QO2Mol_22_36150,QO2Mol_26_128385,QO2Mol_75_108210,QO2Mol_13_140254,QO2Mol_29_22599,QO2Mol_26_73359,QO2Mol_18_63674,QO2Mol_13_28491,QO2Mol_25_39342,QO2Mol_12_189006,QO2Mol_17_12438,QO2Mol_11_151220,QO2Mol_20_176309,QO2Mol_23_46675,QO2Mol_26_124275,QO2Mol_3_75250,QO2Mol_22_150470,QO2Mol_16_85997,QO2Mol_10_108934,QO2Mol_24_29850,QO2Mol_24_55040,QO2Mol_28_197461,QO2Mol_26_82744,QO2Mol_3_186841,QO2Mol_13_179237,QO2Mol_21_122119,QO2Mol_16_69095,QO2Mol_21_82125,QO2Mol_28_1569,QO2Mol_28_55445,QO2Mol_27_62995,QO2Mol_26_38092,QO2Mol_16_25292,QO2Mol_9_112647,QO2Mol_19_136819,QO2Mol_28_75216,QO2Mol_18_67516 |
| [O]N([CH]c1ccco1)c1ccccc1 | QO2Mol_22_52996 | QO2Mol_22_52996,QO2Mol_20_863,QO2Mol_87_41875 |
| [O]N1/C(=C/[CH]c2ccccc2)/CCC1 | QO2Mol_23_158711 | QO2Mol_23_158711,QO2Mol_18_77963,QO2Mol_25_139798,QO2Mol_21_37456 |
| O=C1O[N]N[C]1CN1CCCCC1 | QO2Mol_24_158018 | QO2Mol_24_158018 |
| [O]N1N([O])CC1 | QO2Mol_24_76763 | QO2Mol_24_76763,QO2Mol_79_14008,QO2Mol_17_88220,QO2Mol_10_69961,QO2Mol_9_72719 |
| CC[C@@H]1N([O])C=CN1[O] | QO2Mol_26_103453 | QO2Mol_26_103453,QO2Mol_21_122919,QO2Mol_30_145048,QO2Mol_15_13971,QO2Mol_25_176102,QO2Mol_11_156921,QO2Mol_27_31649,QO2Mol_27_91564,QO2Mol_18_69506,QO2Mol_27_45958,QO2Mol_11_182572,QO2Mol_3_23914,QO2Mol_14_55143,QO2Mol_12_106922,QO2Mol_26_187227,QO2Mol_17_175396,QO2Mol_14_147174,QO2Mol_27_136849,QO2Mol_22_151869,QO2Mol_18_201699,QO2Mol_4_45374,QO2Mol_19_95249,QO2Mol_24_103367,QO2Mol_19_133837,QO2Mol_28_36411,QO2Mol_16_152593,QO2Mol_18_115436,QO2Mol_10_102418,QO2Mol_13_17371,QO2Mol_22_103191,QO2Mol_19_195569,QO2Mol_15_123831,QO2Mol_71_69377,QO2Mol_23_31462,QO2Mol_12_149164,QO2Mol_10_186588,QO2Mol_22_154389,QO2Mol_9_158380,QO2Mol_13_35834,QO2Mol_9_12917,QO2Mol_27_150007,QO2Mol_23_58913,QO2Mol_26_168086,QO2Mol_61_140911,QO2Mol_23_128450,QO2Mol_13_72142,QO2Mol_20_12349,QO2Mol_24_190516,QO2Mol_29_201883,QO2Mol_23_169436,QO2Mol_15_87203,QO2Mol_3_129947,QO2Mol_28_183823,QO2Mol_11_155075,QO2Mol_16_108334,QO2Mol_23_182862,QO2Mol_14_183202,QO2Mol_28_84928,QO2Mol_2_121595,QO2Mol_23_21637,QO2Mol_3_109589,QO2Mol_23_145066,QO2Mol_20_70757,QO2Mol_22_66332,QO2Mol_26_119417,QO2Mol_14_53468,QO2Mol_14_80821,QO2Mol_28_86228,QO2Mol_22_16537,QO2Mol_19_107212,QO2Mol_19_125210,QO2Mol_29_199059,QO2Mol_23_167703,QO2Mol_28_123933,QO2Mol_17_59992,QO2Mol_24_195102,QO2Mol_8_192130,QO2Mol_23_178687,QO2Mol_76_98540,QO2Mol_13_19712,QO2Mol_21_134467,QO2Mol_23_65778,QO2Mol_2_120291,QO2Mol_10_186743,QO2Mol_14_176351,QO2Mol_13_58507 |
| O=C1N[CH][N+]([O-])c2ccccc12 | QO2Mol_29_147833 | QO2Mol_29_147833 |
| [O]N([CH]c1ccccc1)C1CCCCC1 | QO2Mol_31_107793 | QO2Mol_31_107793 |
| O/N=C/1\C=C/C(=C\c2ccc(Cl)cc2)/C=C1 | QO2Mol_32_196094 | QO2Mol_32_196094,QO2Mol_32_191064,QO2Mol_32_195890,QO2Mol_32_195159,QO2Mol_32_193783,QO2Mol_32_194792,QO2Mol_32_191081,QO2Mol_32_194664,QO2Mol_32_197685,QO2Mol_32_193279,QO2Mol_32_191811,QO2Mol_32_194377,QO2Mol_32_195053,QO2Mol_32_197359,QO2Mol_32_191283,QO2Mol_32_192773,QO2Mol_32_191461,QO2Mol_32_199084,QO2Mol_32_199608,QO2Mol_32_197979,QO2Mol_32_195367,QO2Mol_32_196857,QO2Mol_32_191416,QO2Mol_32_197850,QO2Mol_32_196763,QO2Mol_32_199409,QO2Mol_32_197797,QO2Mol_32_198425,QO2Mol_32_191458,QO2Mol_32_191691,QO2Mol_32_191545,QO2Mol_32_194478,QO2Mol_32_191031,QO2Mol_32_197523,QO2Mol_32_196996,QO2Mol_32_197785,QO2Mol_32_196162,QO2Mol_32_199183,QO2Mol_32_198441,QO2Mol_32_195875,QO2Mol_32_198220,QO2Mol_32_197215,QO2Mol_32_191488,QO2Mol_32_195186,QO2Mol_32_192323,QO2Mol_32_199578,QO2Mol_32_193922,QO2Mol_32_197476,QO2Mol_32_192289,QO2Mol_32_198431,QO2Mol_32_197017,QO2Mol_32_195788,QO2Mol_32_196214,QO2Mol_32_196470,QO2Mol_32_194411,QO2Mol_32_193915,QO2Mol_32_196196,QO2Mol_32_196809,QO2Mol_32_196878,QO2Mol_32_192099,QO2Mol_32_194238,QO2Mol_32_191530,QO2Mol_32_193295,QO2Mol_32_193530,QO2Mol_32_197191,QO2Mol_32_191124,QO2Mol_32_197590,QO2Mol_32_193891,QO2Mol_32_194703,QO2Mol_32_194177,QO2Mol_32_194089,QO2Mol_32_194532,QO2Mol_32_197143,QO2Mol_32_196333,QO2Mol_32_199570,QO2Mol_32_192654,QO2Mol_32_193177,QO2Mol_32_191553,QO2Mol_32_195015 |
| O/N=C/1\C=C[C]([CH]c2ccc(Cl)cc2)C=C1 | QO2Mol_32_198158 | QO2Mol_32_198158 |
| O=CN/N=C/1\C=C/C(=N/O)/C=C1 | QO2Mol_37_116839 | QO2Mol_37_116839,QO2Mol_48_180808 |
| [O]N1N([O])[C@H](c2ccccc2)C1 | QO2Mol_38_120618 | QO2Mol_38_120618,QO2Mol_46_118318,QO2Mol_58_141674,QO2Mol_82_164918,QO2Mol_66_180037,QO2Mol_31_182874,QO2Mol_47_64539,QO2Mol_48_119905,QO2Mol_59_48042,QO2Mol_43_116538,QO2Mol_78_188342,QO2Mol_82_71679,QO2Mol_39_90380,QO2Mol_52_48684,QO2Mol_32_152970,QO2Mol_56_199302,QO2Mol_40_14644,QO2Mol_74_93684,QO2Mol_79_8588,QO2Mol_73_80976,QO2Mol_70_107515,QO2Mol_61_78287,QO2Mol_45_141511,QO2Mol_67_132775,QO2Mol_87_176561,QO2Mol_58_145168,QO2Mol_31_21724,QO2Mol_60_4273,QO2Mol_58_194636,QO2Mol_83_34960,QO2Mol_58_35948,QO2Mol_47_117228,QO2Mol_77_170688,QO2Mol_84_127018,QO2Mol_44_180986 |
| [O-][N@+]1[CH][C](c2ccccc2)[N@+]([O-])CC1 | QO2Mol_38_26677 | QO2Mol_38_26677,QO2Mol_52_18373,QO2Mol_38_192218,QO2Mol_57_156235,QO2Mol_54_113550,QO2Mol_69_58913,QO2Mol_38_64369,QO2Mol_79_7675,QO2Mol_72_174800,QO2Mol_73_67875,QO2Mol_47_145688,QO2Mol_41_152856,QO2Mol_51_91529,QO2Mol_42_96337,QO2Mol_48_53407,QO2Mol_32_6349,QO2Mol_52_169893,QO2Mol_80_50774,QO2Mol_44_174689,QO2Mol_79_81418,QO2Mol_58_11437,QO2Mol_38_179853,QO2Mol_39_65959, QO2Mol_60_84791,QO2Mol_44_9437,QO2Mol_73_40554,QO2Mol_67_78427,QO2Mol_68_57544,QO2Mol_62_16534,QO2Mol_56_27830,QO2Mol_63_577,QO2Mol_86_149346,QO2Mol_64_103123,QO2Mol_75_172313,QO2Mol_59_18334,QO2Mol_85_38680,QO2Mol_44_1194,QO2Mol_38_36652,QO2Mol_37_95650,QO2Mol_82_180874,QO2Mol_37_170020,QO2Mol_56_166207 |
| O/N=C/1\C=C/C(=N\O)/C=C1 | QO2Mol_38_2675 | QO2Mol_38_2675,QO2Mol_87_52684,QO2Mol_47_142625,QO2Mol_45_159761,QO2Mol_79_76223,QO2Mol_54_80049,QO2Mol_71_158702,QO2Mol_56_99405,QO2Mol_69_141110,QO2Mol_89_1326,QO2Mol_81_61767,QO2Mol_47_40418,QO2Mol_56_106805,QO2Mol_50_201513 |
| [O]N1C=C(c2ccc(Cl)cc2)N([O])C1 | QO2Mol_39_135886 | QO2Mol_39_135886,QO2Mol_78_163471,QO2Mol_73_138450,QO2Mol_58_162653,QO2Mol_62_27139,QO2Mol_48_19710,QO2Mol_52_64536,QO2Mol_81_201233,QO2Mol_57_184547,QO2Mol_47_115634,QO2Mol_41_136634,QO2Mol_57_73833,QO2Mol_77_179221,QO2Mol_45_29238,QO2Mol_57_11221,QO2Mol_68_26645,QO2Mol_50_15014,QO2Mol_56_171783,QO2Mol_82_175433,QO2Mol_82_128674,QO2Mol_70_137527,QO2Mol_69_87949,QO2Mol_72_155080,QO2Mol_54_18265,QO2Mol_37_104824,QO2Mol_42_190507,QO2Mol_58_30487,QO2Mol_63_176797,QO2Mol_50_197764,QO2Mol_84_130379,QO2Mol_42_74473,QO2Mol_52_73364,QO2Mol_40_5461,QO2Mol_66_95735 |
| N#C/C=C\1/C=C/C(=N/O)/C=C1Cl | QO2Mol_39_56214 | QO2Mol_39_56214,QO2Mol_50_173711,QO2Mol_76_132891,QO2Mol_59_31906 |
| CN1C=C([NH])O[N]1 | QO2Mol_4_106170 | QO2Mol_4_106170,QO2Mol_13_58715,QO2Mol_17_13060,QO2Mol_26_170704,QO2Mol_25_88060,QO2Mol_17_74822,QO2Mol_30_133201,QO2Mol_18_156931,QO2Mol_29_142962,QO2Mol_13_749,QO2Mol_29_39104,QO2Mol_23_40530,QO2Mol_88_90869 |
| [O]N1C=C(c2cccs2)N([O])C1 | QO2Mol_45_145277 | QO2Mol_45_145277,QO2Mol_60_193587,QO2Mol_81_46978,QO2Mol_37_186043,QO2Mol_67_36628,QO2Mol_44_36441,QO2Mol_46_179162,QO2Mol_80_111447,QO2Mol_54_91964,QO2Mol_58_132467,QO2Mol_59_46730,QO2Mol_64_182508,QO2Mol_57_201695,QO2Mol_41_123641,QO2Mol_56_70563,QO2Mol_32_108412,QO2Mol_65_57620,QO2Mol_67_64162,QO2Mol_82_39612,QO2Mol_30_194284,QO2Mol_87_196470,QO2Mol_44_100364,QO2Mol_57_138824,QO2Mol_46_148835,QO2Mol_75_179127,QO2Mol_81_47571,QO2Mol_63_34041,QO2Mol_41_21884,QO2Mol_80_29258,QO2Mol_67_122841,QO2Mol_38_121619,QO2Mol_53_101234,QO2Mol_66_1075,QO2Mol_59_46167,QO2Mol_58_174862,QO2Mol_80_73531,QO2Mol_47_84846,QO2Mol_63_147780 |
| [O]N1ON=C2C=CC=C[C]12 | QO2Mol_45_58938 | QO2Mol_45_58938,QO2Mol_80_198323,QO2Mol_46_133337 |
| C[C@H]1CN([O])N1[O] | QO2Mol_46_14220 | QO2Mol_46_14220,QO2Mol_50_46480,QO2Mol_86_120775 |
| N#C/C=C/1\C=C/C(=N\O)/C=C1 | QO2Mol_48_117155 | QO2Mol_48_117155,QO2Mol_53_114355,QO2Mol_54_40736,QO2Mol_40_8743,QO2Mol_54_200449,QO2Mol_37_119953,QO2Mol_39_106814,QO2Mol_65_131846,QO2Mol_85_41842,QO2Mol_41_171193,QO2Mol_84_162344,QO2Mol_36_155868,QO2Mol_43_109299,QO2Mol_31_83599,QO2Mol_71_54868,QO2Mol_65_171782,QO2Mol_51_118582,QO2Mol_88_175742,QO2Mol_63_33113,QO2Mol_61_98892,QO2Mol_32_11276,QO2Mol_40_201879,QO2Mol_44_69377,QO2Mol_51_155086,QO2Mol_65_10583,QO2Mol_85_68594 |
| C[C@@H]1C[N+]([O-])C=C[N+]1[O-] | QO2Mol_5_115152 | QO2Mol_5_115152,QO2Mol_5_121375,QO2Mol_5_120567,QO2Mol_5_120178,QO2Mol_5_119484,QO2Mol_5_119047,QO2Mol_5_114871,QO2Mol_5_118781,QO2Mol_5_121899,QO2Mol_5_114615,QO2Mol_5_120285,QO2Mol_5_115615,QO2Mol_5_113497,QO2Mol_5_121203,QO2Mol_5_113832,QO2Mol_5_116719,QO2Mol_5_121257,QO2Mol_5_115410,QO2Mol_5_115593,QO2Mol_5_113388 |
| O=C1OC=N/C/1=C\C(=C\c1ccccc1)\Cl | QO2Mol_51_105355 | QO2Mol_51_105355 |
| N/N=C\1/C=C/C(=N/O)/C=C1 | QO2Mol_51_158784 | QO2Mol_51_158784,QO2Mol_68_185014,QO2Mol_51_40368,QO2Mol_45_129602 |
| [O-][n+]1cn[n+]([O-])c2ccccc12 | QO2Mol_51_22214 | QO2Mol_51_22214,QO2Mol_54_97484,QO2Mol_51_165386,QO2Mol_50_133958,QO2Mol_30_191859,QO2Mol_68_149140 |
| [O]N1C=NN([O])c2cnccc12 | QO2Mol_51_86018 | QO2Mol_51_86018 |
| [O-][n+]1cc[n+]([O-])c2cc(Cl)ccc12 | QO2Mol_54_113937 | QO2Mol_54_113937,QO2Mol_55_177173,QO2Mol_87_24383 |
| O=CN/N=C/1\C=C/C(=N/OC(=O)c2ccccc2)/C=C1 | QO2Mol_55_53886 | QO2Mol_55_53886,QO2Mol_83_181321,QO2Mol_68_79343,QO2Mol_54_146724,QO2Mol_47_85115,QO2Mol_83_116288,QO2Mol_86_151260,QO2Mol_53_157846,QO2Mol_31_133622 |
| [NH]C1=CN(Cc2ccccc2)[N]O1 | QO2Mol_56_161095 | QO2Mol_56_161095,QO2Mol_52_191468,QO2Mol_45_43995,QO2Mol_46_190468,QO2Mol_83_181683,QO2Mol_76_163455,QO2Mol_65_122791,QO2Mol_55_10696,QO2Mol_80_16962,QO2Mol_68_134931,QO2Mol_75_27350,QO2Mol_82_173991,QO2Mol_79_134285,QO2Mol_55_105337,QO2Mol_65_31588,QO2Mol_78_157806,QO2Mol_37_148323,QO2Mol_50_52017,QO2Mol_40_182998,QO2Mol_68_165021,QO2Mol_69_126237,QO2Mol_87_24241 |
| O/N=C/1\C=CC(=C\C1=N/O)/C=C/c1ccccc1 | QO2Mol_56_55210 | QO2Mol_56_55210,QO2Mol_59_100369,QO2Mol_38_194814,QO2Mol_74_34430 |
| N#C/C=C\1/C=C(Br)/C(=N/O)/C=C1Cl | QO2Mol_59_143043 | QO2Mol_59_143043,QO2Mol_82_107006,QO2Mol_43_90095 |
| [O-][n+]1cn[n+]([O-])c2cnccc12 | QO2Mol_59_189155 | QO2Mol_59_189155,QO2Mol_48_34735,QO2Mol_50_4535 |
| N=C(N)N[N]c1ccc([N]NC(=S)N)cc1 | QO2Mol_61_112924 | QO2Mol_61_112924,QO2Mol_44_99493,QO2Mol_70_175774,QO2Mol_73_135437,QO2Mol_48_6756 |
| [O-][N+]1C=C[N+]([O-])[C@@H]2CCCC[C@@H]12 | QO2Mol_62_126012 | QO2Mol_62_126012,QO2Mol_42_158786,QO2Mol_51_16891 |
| O=C1OC=N/C/1=C/c1ccco1 | QO2Mol_62_34581 | QO2Mol_62_34581,QO2Mol_62_174880 |
| [O-][n+]1cc[n+]([O-])c2ccccc12 | QO2Mol_64_48480 | QO2Mol_64_48480,QO2Mol_39_44492,QO2Mol_41_108785,QO2Mol_29_172490,QO2Mol_52_4624,OE62_34032 |
| [O-][N+]1C=C[N+]([O-])CC1 | QO2Mol_66_140534 | QO2Mol_66_140534,QO2Mol_62_95676,QO2Mol_39_71750,QO2Mol_71_88482,QO2Mol_52_60543,QO2Mol_31_118777 |
| CCCC(N=[N])N=[N] | QO2Mol_66_156961 | QO2Mol_66_156961 |
| [O]N1N([O])[C@H](O)C1 | QO2Mol_66_62492 | QO2Mol_66_62492,QO2Mol_42_129388,QO2Mol_81_150209,QO2Mol_42_34227,QO2Mol_47_154439,QO2Mol_46_138959,QO2Mol_32_118903,QO2Mol_66_36346,QO2Mol_70_133903,QO2Mol_52_78733,QO2Mol_39_124462,QO2Mol_42_135022,QO2Mol_52_134283,QO2Mol_89_94558,QO2Mol_74_47780,QO2Mol_45_104924,QO2Mol_62_97209,QO2Mol_73_35599,QO2Mol_65_169726,QO2Mol_64_106928,QO2Mol_73_36386,QO2Mol_67_12561,QO2Mol_55_148196,QO2Mol_84_4459,QO2Mol_84_492,QO2Mol_70_199058,QO2Mol_41_94095,QO2Mol_86_176187,QO2Mol_89_39675,QO2Mol_54_51063,QO2Mol_88_42313,QO2Mol_57_154289,QO2Mol_45_83270,QO2Mol_56_40670,QO2Mol_47_191184,QO2Mol_42_170774,QO2Mol_42_193830 |
| [NH]C1=CN(c2ccccc2)[N]O1 | QO2Mol_7_14236 | QO2Mol_7_14236,QO2Mol_7_17042,QO2Mol_7_12264,QO2Mol_7_10950,QO2Mol_7_15671,QO2Mol_7_13170,QO2Mol_7_16394,QO2Mol_7_17089,QO2Mol_7_14863,QO2Mol_7_13500,QO2Mol_7_15359,QO2Mol_7_11760,QO2Mol_7_15369,QO2Mol_7_14500,QO2Mol_7_15492,QO2Mol_7_12980,QO2Mol_7_10452,QO2Mol_7_9940,QO2Mol_7_14505,QO2Mol_7_13951,QO2Mol_7_10467,QO2Mol_7_13444,QO2Mol_7_10503,QO2Mol_7_13096,QO2Mol_7_11576,QO2Mol_7_10447,QO2Mol_7_11339,QO2Mol_7_15661,QO2Mol_7_17367,QO2Mol_7_14781,QO2Mol_7_11934,QO2Mol_7_14564,QO2Mol_7_16330,QO2Mol_7_17386,QO2Mol_7_12093,QO2Mol_7_11359,QO2Mol_7_12363,QO2Mol_7_12806,QO2Mol_7_12969,QO2Mol_7_11627,QO2Mol_7_10148,QO2Mol_7_17416,QO2Mol_7_18117,QO2Mol_7_14570,QO2Mol_7_16986,QO2Mol_7_13017 |
| O/N=C/1\C=Cc2[nH]ccc2\C1=N/O | QO2Mol_70_110625 | QO2Mol_70_110625 |
| C[C@H]1N([O])C=CN1[O] | QO2Mol_70_122680 | QO2Mol_70_122680,QO2Mol_41_9750,QO2Mol_32_81166 |
| [O]N1C=C(c2ccccc2)N([O])C1 | QO2Mol_71_162667 | QO2Mol_71_162667,QO2Mol_59_185963,QO2Mol_70_46210,QO2Mol_56_137010,QO2Mol_45_121417,QO2Mol_42_63730,QO2Mol_63_135404,QO2Mol_37_187578,QO2Mol_39_140432,QO2Mol_54_151945,QO2Mol_55_196985,QO2Mol_38_33547,QO2Mol_51_73623,QO2Mol_85_69565,QO2Mol_53_114649,QO2Mol_43_163561,QO2Mol_76_27724,QO2Mol_64_53554,QO2Mol_51_136471,QO2Mol_57_58317,QO2Mol_66_37200,QO2Mol_54_147900,QO2Mol_56_81537,QO2Mol_66_154549 |
| CC1=C/C(=N\O)/C(=N/O)/C=C1 | QO2Mol_73_180416 | QO2Mol_73_180416,QO2Mol_73_99101,QO2Mol_32_4039,QO2Mol_86_57452,QO2Mol_86_41444,QO2Mol_46_130586,QO2Mol_55_117062,QO2Mol_45_107797,QO2Mol_55_25553,QO2Mol_69_112077,QO2Mol_31_145639,QO2Mol_64_109669,QO2Mol_42_16035,QO2Mol_77_21076,QO2Mol_77_165263,QO2Mol_51_122303,QO2Mol_52_199747,QO2Mol_41_130517,QO2Mol_69_128047,QO2Mol_88_2747,QO2Mol_85_81134,QO2Mol_50_30311 |
| CC(C)NC(=O)N[N][O] | QO2Mol_74_57021 | QO2Mol_74_57021 |
| COC(=O)[N]C1=CN[N]O1 | QO2Mol_76_78707 | QO2Mol_76_78707,QO2Mol_73_91662,QO2Mol_58_60574,QO2Mol_51_19095,QO2Mol_51_91524,QO2Mol_37_196833,QO2Mol_45_118512,QO2Mol_51_119487,QO2Mol_47_137365,QO2Mol_36_193125,QO2Mol_51_30267,QO2Mol_76_147642,QO2Mol_65_185654,QO2Mol_44_134535,QO2Mol_63_114986,QO2Mol_63_96345,QO2Mol_48_180638 |
| NC(=O)[N]C1=CN[N]O1 | QO2Mol_76_82130 | QO2Mol_76_82130,QO2Mol_71_155644,QO2Mol_61_73150,QO2Mol_50_163810,QO2Mol_72_65020,QO2Mol_39_88200 |
| O=C(O)[N]C1=CN[N]O1 | QO2Mol_78_123263 | QO2Mol_78_123263,QO2Mol_45_75597,QO2Mol_66_5773,QO2Mol_70_63334,QO2Mol_52_50983,QO2Mol_45_156692,QO2Mol_79_79843,QO2Mol_70_20503,QO2Mol_58_138391,QO2Mol_59_162230,QO2Mol_52_130080,QO2Mol_77_91322,QO2Mol_56_168033,QO2Mol_39_5872,QO2Mol_68_184563,QO2Mol_83_185147,QO2Mol_54_134876,QO2Mol_82_140784,QO2Mol_74_56562,QO2Mol_45_63737,QO2Mol_39_559,QO2Mol_56_136757,QO2Mol_82_138824,QO2Mol_89_31742,QO2Mol_75_177156,QO2Mol_71_161572,QO2Mol_82_97439,QO2Mol_44_148091,QO2Mol_74_135272,QO2Mol_76_93143,QO2Mol_85_42694,QO2Mol_61_25827,QO2Mol_58_110579,QO2Mol_71_75478,QO2Mol_66_189345,QO2Mol_59_31695,QO2Mol_85_20839,QO2Mol_50_62146,QO2Mol_31_46892,QO2Mol_69_55676,QO2Mol_55_198720,QO2Mol_73_78465,QO2Mol_87_122725,QO2Mol_87_165011,QO2Mol_88_88628,QO2Mol_59_153807,QO2Mol_82_87190,QO2Mol_58_145324,QO2Mol_67_137708,QO2Mol_30_180115,QO2Mol_64_117335,QO2Mol_57_86091,QO2Mol_84_183957,QO2Mol_51_200814,QO2Mol_32_14334,QO2Mol_57_115918,QO2Mol_39_32710,QO2Mol_31_78038,QO2Mol_51_147848,QO2Mol_68_30143,QO2Mol_55_114648,QO2Mol_42_612,QO2Mol_68_45316,QO2Mol_31_119417,QO2Mol_66_162881,QO2Mol_38_116571,QO2Mol_42_101701,QO2Mol_47_37496,QO2Mol_50_90693,QO2Mol_81_167917,QO2Mol_42_184935,QO2Mol_66_79987,QO2Mol_56_47844,QO2Mol_32_45356,QO2Mol_52_194553 |
| CCN1[CH]C(=O)O[N]1 | QO2Mol_78_56404 | QO2Mol_78_56404,QO2Mol_43_58054 |
| [O]N1C2=C(CCCC2)N([O])C1 | QO2Mol_78_79089 | QO2Mol_78_79089,QO2Mol_76_125827,QO2Mol_47_88697,QO2Mol_75_175187,QO2Mol_57_28049,QO2Mol_65_76890,QO2Mol_41_137968,QO2Mol_42_176138 |
| O=C1OC=N/C/1=C/c1ccc[nH]1 | QO2Mol_79_161609 | QO2Mol_79_161609,QO2Mol_73_44657 |
| O=CN/N=C\1/C=C/C(=N/OC=O)/C=C1 | QO2Mol_79_43660 | QO2Mol_79_43660,QO2Mol_51_160217,QO2Mol_83_154041,QO2Mol_74_43616 |
| CN([O])[CH]c1ccc[nH]1 | QO2Mol_80_170100 | QO2Mol_80_170100,QO2Mol_64_89529,QO2Mol_85_7703,QO2Mol_83_129734,QO2Mol_64_102342,QO2Mol_82_93910 |
| N=C(N)N[N]c1ccc([N]NC(=N)N)cc1 | QO2Mol_80_53686 | QO2Mol_80_53686,QO2Mol_45_183057,QO2Mol_42_99297 |
| CCN1C=C([NH])O[N]1 | QO2Mol_82_44744 | QO2Mol_82_44744,QO2Mol_55_55102,QO2Mol_47_182080,QO2Mol_81_59413,QO2Mol_71_172656,QO2Mol_83_32238,QO2Mol_41_26771,QO2Mol_54_63822,QO2Mol_38_57158,QO2Mol_75_25422,QO2Mol_41_189578,QO2Mol_58_125119,QO2Mol_89_65732,QO2Mol_52_188443,QO2Mol_89_48216,QO2Mol_43_198579,QO2Mol_85_169963,QO2Mol_62_186264,QO2Mol_42_112599,QO2Mol_37_202214,QO2Mol_70_102306,QO2Mol_67_96440,QO2Mol_41_165377,QO2Mol_40_100347,QO2Mol_61_101678,QO2Mol_50_70516,QO2Mol_65_64602,QO2Mol_55_116439,QO2Mol_32_79167,QO2Mol_41_9182,QO2Mol_55_7167,QO2Mol_75_90873,QO2Mol_40_179887,QO2Mol_77_65763,QO2Mol_43_132393,QO2Mol_46_37561,QO2Mol_51_49443,QO2Mol_80_126549,QO2Mol_48_198455,QO2Mol_76_114319,QO2Mol_61_163079,QO2Mol_82_89359,QO2Mol_38_140592,QO2Mol_55_190324,QO2Mol_82_137847,QO2Mol_53_54744,QO2Mol_77_201521,QO2Mol_50_132520,QO2Mol_89_59375,QO2Mol_58_50152,QO2Mol_70_93310,QO2Mol_37_77501,QO2Mol_63_107464,QO2Mol_32_124435,QO2Mol_53_173119,QO2Mol_77_168032,QO2Mol_82_3715,QO2Mol_39_49384 |
| [O]N([CH]c1ccco1)C1CCCCC1 | QO2Mol_83_190900 | QO2Mol_83_190900,QO2Mol_71_156859,QO2Mol_40_64577,QO2Mol_85_172004,QO2Mol_51_96651,QO2Mol_66_4648,QO2Mol_84_70675,QO2Mol_47_15611 |
| CC(=O)O/N=C\1/C=C/C(=C\C#N)/C=C1 | QO2Mol_84_24814 | QO2Mol_84_24814,QO2Mol_44_105407,QO2Mol_50_150626,QO2Mol_63_137256,QO2Mol_48_30955,QO2Mol_54_184835,QO2Mol_61_14176,QO2Mol_45_199915,QO2Mol_52_116545,QO2Mol_63_154869,QO2Mol_47_49875,QO2Mol_82_6537,QO2Mol_57_69893,QO2Mol_85_50793,QO2Mol_43_185270,QO2Mol_79_116897,QO2Mol_64_196066,QO2Mol_85_71084,QO2Mol_77_25672,QO2Mol_67_186864,QO2Mol_39_147852,QO2Mol_47_129643,QO2Mol_51_24922,QO2Mol_65_19045,QO2Mol_81_185957,QO2Mol_40_152666,QO2Mol_84_24027,QO2Mol_70_130107,QO2Mol_36_173060,QO2Mol_77_143566,QO2Mol_42_101448,QO2Mol_43_140370,QO2Mol_42_71256,QO2Mol_40_198658,QO2Mol_47_31012 |
| CC1=CN(CN1[O])[O] | QO2Mol_86_103397 | QO2Mol_86_103397,QO2Mol_75_120951,QO2Mol_52_23406,QO2Mol_40_199589,QO2Mol_65_146854,QO2Mol_80_161150,QO2Mol_64_111336,QO2Mol_86_13341,QO2Mol_52_70779,QO2Mol_38_171989,QO2Mol_69_195396,QO2Mol_68_111032,QO2Mol_54_59479,QO2Mol_43_9886 |
| [O]N1ON=C2[CH]C=CC=C12 | QO2Mol_89_99619 | QO2Mol_89_99619,QO2Mol_87_139188 |

Table S3. Summary of 79 easily-synthesizable SF molecules including filename, *E*_S1_, *E*_T1_, *E*_T2_, SF score at GW+BSE level and their DeepSA score.

| Filename | *E*_S1_ | *E*_T1_ | *E*_T2_ | SF score | DeepSA score |
| --- | --- | --- | --- | --- | --- |
| OE62_2436 | 4.10 | 1.62 | 3.87 | 0.53 | 0.75 |
| OE62_55804 | 4.16 | 1.69 | 3.91 | 0.41 | 0.57 |
| QO2Mol_64_48480 | 3.65 | 1.31 | 3.00 | 0.38 | 0.84 |
| QO2Mol_54_113937 | 3.63 | 1.29 | 2.95 | 0.38 | 0.83 |
| QO2Mol_38_26677 | 3.73 | 1.47 | 3.34 | 0.32 | 0.81 |
| QO2Mol_51_22214 | 3.48 | 1.27 | 2.83 | 0.28 | 0.85 |
| QO2Mol_59_189155 | 3.40 | 1.22 | 2.70 | 0.25 | 0.84 |
| OE62_17368 | 3.39 | 1.42 | 3.27 | 0.23 | 0.83 |
| OE62_60610 | 3.83 | 1.49 | 3.25 | 0.22 | 0.75 |
| QO2Mol_71_162667 | 3.58 | 1.48 | 3.25 | 0.18 | 0.51 |
| OE62_31066 | 3.62 | 1.52 | 3.33 | 0.17 | 0.83 |
| OE62_1627 | 4.22 | 1.57 | 3.29 | 0.16 | 0.57 |
| OE62_48111 | 3.40 | 1.45 | 3.22 | 0.16 | 0.69 |
| QO2Mol_48_117155 | 4.51 | 1.87 | 3.93 | 0.15 | 0.65 |
| OE62_42482 | 3.72 | 1.60 | 3.48 | 0.14 | 0.75 |
| OE62_57269 | 3.86 | 1.55 | 3.26 | 0.12 | 0.70 |
| OE62_6193 | 4.36 | 1.92 | 3.98 | 0.07 | 0.97 |
| OE62_1014 | 4.79 | 2.22 | 4.58 | 0.05 | 0.87 |
| QO2Mol_20_197704 | 4.03 | 1.62 | 3.30 | 0.05 | 0.54 |
| OE62_42621 | 3.66 | 1.58 | 3.26 | 0.04 | 0.58 |
| QO2Mol_66_140534 | 3.76 | 1.34 | 2.72 | 0.04 | 0.51 |
| QO2Mol_61_112924 | 3.52 | 1.58 | 3.24 | 0.03 | 0.69 |
| OE62_46747 | 3.63 | 1.54 | 3.13 | 0.03 | 0.68 |
| OE62_23043 | 4.02 | 1.89 | 3.87 | 0.02 | 0.66 |
| OE62_9458 | 3.79 | 1.55 | 3.13 | 0.02 | 0.81 |
| OE62_23772 | 3.47 | 1.45 | 2.92 | 0.01 | 0.71 |
| OE62_58106 | 3.35 | 1.50 | 3.03 | 0.01 | 0.76 |
| QO2Mol_73_180416 | 4.01 | 1.69 | 3.38 | 0.00 | 0.71 |
| OE62_23194 | 3.46 | 1.57 | 3.14 | 0.00 | 0.56 |
| QO2Mol_51_158784 | 3.49 | 1.68 | 3.37 | 0.00 | 0.54 |
| QO2Mol_38_2675 | 4.38 | 2.05 | 4.09 | -0.01 | 0.60 |
| OE62_48647 | 4.15 | 1.88 | 3.73 | -0.02 | 0.57 |
| OE62_38058 | 4.46 | 2.14 | 4.25 | -0.03 | 1.00 |
| OE62_39434 | 4.71 | 2.10 | 4.18 | -0.03 | 0.69 |
| OE62_45594 | 3.66 | 1.65 | 3.24 | -0.05 | 0.49 |
| OE62_8345 | 4.06 | 1.94 | 3.82 | -0.06 | 0.99 |
| OE62_48887 | 3.62 | 1.45 | 2.85 | -0.06 | 0.99 |
| OE62_36154 | 3.67 | 1.72 | 3.34 | -0.11 | 0.72 |
| OE62_46183 | 3.56 | 1.47 | 2.83 | -0.12 | 0.99 |
| OE62_51529 | 3.75 | 1.72 | 3.32 | -0.12 | 0.51 |
| OE62_50355 | 4.28 | 2.03 | 3.91 | -0.15 | 0.78 |
| QO2Mol_56_161095 | 4.18 | 2.16 | 4.15 | -0.16 | 0.64 |
| OE62_59527 | 2.93 | 1.39 | 2.62 | -0.17 | 0.81 |
| QO2Mol_80_53686 | 3.50 | 1.80 | 3.43 | -0.17 | 0.54 |
| OE62_53890 | 3.43 | 1.78 | 3.38 | -0.18 | 0.68 |
| QO2Mol_79_161609 | 4.12 | 2.11 | 4.03 | -0.20 | 0.74 |
| OE62_40747 | 3.85 | 1.93 | 3.65 | -0.22 | 1.00 |
| QO2Mol_12_153647 | 4.10 | 2.12 | 3.99 | -0.26 | 0.88 |
| OE62_26595 | 3.77 | 1.72 | 3.18 | -0.26 | 0.85 |
| OE62_18562 | 3.51 | 1.73 | 3.17 | -0.29 | 0.78 |
| QO2Mol_7_14236 | 3.60 | 1.91 | 3.52 | -0.29 | 0.54 |
| OE62_53903 | 4.07 | 1.97 | 3.62 | -0.32 | 1.00 |
| OE62_345 | 3.98 | 2.15 | 3.96 | -0.35 | 0.53 |
| QO2Mol_15_184828 | 3.84 | 2.04 | 3.62 | -0.47 | 0.98 |
| QO2Mol_23_158711 | 3.84 | 2.05 | 3.62 | -0.47 | 0.97 |
| OE62_22523 | 4.17 | 1.92 | 3.37 | -0.47 | 0.50 |
| QO2Mol_38_120618 | 4.26 | 2.35 | 4.20 | -0.50 | 0.52 |
| QO2Mol_22_52996 | 3.73 | 2.07 | 3.63 | -0.50 | 0.98 |
| OE62_4847 | 2.59 | 1.52 | 2.52 | -0.51 | 0.55 |
| QO2Mol_70_110625 | 3.61 | 1.91 | 3.27 | -0.55 | 0.52 |
| QO2Mol_51_105355 | 4.13 | 2.20 | 3.84 | -0.55 | 0.48 |
| QO2Mol_62_34581 | 4.22 | 2.26 | 3.92 | -0.60 | 0.75 |
| OE62_34944 | 3.72 | 1.80 | 2.99 | -0.61 | 0.69 |
| QO2Mol_31_107793 | 3.85 | 2.07 | 3.53 | -0.62 | 0.99 |
| QO2Mol_15_100540 | 3.36 | 1.86 | 3.06 | -0.67 | 0.95 |
| OE62_8326 | 3.77 | 1.94 | 3.14 | -0.74 | 0.67 |
| OE62_5505 | 3.91 | 2.16 | 3.55 | -0.76 | 0.50 |
| OE62_25261 | 3.59 | 1.91 | 3.05 | -0.76 | 0.82 |
| QO2Mol_83_190900 | 3.71 | 2.23 | 3.68 | -0.79 | 0.97 |
| OE62_11827 | 3.85 | 2.31 | 3.82 | -0.80 | 0.65 |
| QO2Mol_78_56404 | 4.39 | 2.74 | 4.32 | -1.17 | 0.72 |
| QO2Mol_24_76763 | 4.06 | 2.60 | 4.01 | -1.19 | 0.56 |
| QO2Mol_76_78707 | 4.23 | 2.72 | 4.18 | -1.26 | 0.54 |
| QO2Mol_78_123263 | 4.35 | 2.83 | 4.30 | -1.36 | 0.48 |
| QO2Mol_74_57021 | 3.71 | 2.54 | 4.54 | -1.37 | 0.94 |
| QO2Mol_13_118083 | 3.96 | 2.58 | 3.75 | -1.40 | 0.68 |
| QO2Mol_80_170100 | 3.12 | 2.28 | 3.09 | -1.47 | 0.78 |
| QO2Mol_29_147833 | 3.64 | 2.32 | 3.12 | -1.52 | 0.73 |
| QO2Mol_14_125532 | 3.56 | 2.63 | 3.52 | -1.74 | 0.77 |

**S6. GW+BSE convergence validation**

For all molecular systems, we employed a cubic supercell with a total vacuum thickness of 15 Å (defined as the distance between periodic images). This ensures that the electronic density of the isolated molecule is well-contained within the supercell. To strictly eliminate spurious long-range Coulomb interactions between periodic images, we applied the Coulomb truncation scheme based on the Wigner-Seitz cell boundaries, which is the recommended approach for 0D (molecular) systems to achieve faster convergence with respect to the vacuum size.

To test the convergence validation, we selected Pentacene as an example, which is a well-established benchmark for singlet fission. Table S4 compares the quasiparticle (QP) band gap of pentacene molecule with different kinetic energy cutoffs of the dielectric matrices. While there is still a small discrepancy with regard to experimental data, our test shows that the QP band gap is already converged at a dielectric matrix cutoff of 12 Ry. Further increasing this value does not significantly change the results.

Table S4. The QP band gaps with different kinetic-energy cutoffs of the dielectric matrices.

| Dielectric matrix cutoff (Ry) | 6 | 12 | 18 | 25 | Exp. [11] |
| --- | --- | --- | --- | --- | --- |
| QP band gap (eV) | 4.998 | 5.014 | 5.021 | 5.023 | 5.3 |

In addition, the convergence test with different numbers of bands is shown in Table S5. Considering both precision and cost, 500 bands are sufficient for pentacene, which has 51 valence bands. For other systems in this work, the number of bands is maintained at about **ten times the number of valence band**. The present setup provides sufficient accuracy for evaluating the energetic criterion for singlet fission, which is mainly characterized by the energy levels of excited states.

Table S5. Convergence behavior with different numbers of bands.

| Number of bands | ***E*_S1_** (eV) | ***E*_T1_** (eV) | ***E*_T2_** (eV) | ***E*_S1_-2*E_T_*_1_** (eV) | ***E*_T2_-2*E_T_*_1_** (eV) |
| --- | --- | --- | --- | --- | --- |
| 200 | 2.950 | 1.288 | 2.502 | 0.374 | -0.074 |
| 300 | 2.873 | 1.229 | 2.427 | 0.414 | -0.032 |
| 400 | 2.787 | 1.162 | 2.352 | 0.463 | 0.028 |
| 500 | 2.716 | 1.108 | 2.289 | 0.500 | 0.073 |
| 600 | 2.672 | 1.073 | 2.251 | 0.526 | 0.106 |
| 700 | 2.645 | 1.054 | 2.230 | 0.537 | 0.122 |

Furthermore, we also test the convergence of the energies for excited states with respect to the truncation of the dielectric matrix. As shown in Table S6, the energies of excited levels converge well with 6 valence bands (*N*_v_) and 6 conduction bands (*N*_c_) across dielectric matrix cutoffs of 6, 12, and 18 Ry. A cutoff of 12 Ry with *N*_v_, *N*_c_ = 6 was selected for subsequent calculations to balance numerical accuracy and computational efficiency.

Table S6. Convergence behavior of the energies of excited levels.

| Dielectric matrix cut-off (Ry) | ***N*_v_** | ***N*_c_** | ***E*_S1_** (eV) | ***E*_T1_** (eV) | ***E*_T2_** (eV) |
| --- | --- | --- | --- | --- | --- |
| 6 | 3 | 4 | 2.850 | 1.134 | 2.370 |
|  | 6 | 6 | 2.706 | 1.086 | 2.269 |
|  | 8 | 5 | 2.708 | 1.086 | 2.266 |
|  | 8 | 8 | 2.631 | 1.067 | 2.250 |
|  | 10 | 10 | 2.631 | 1.067 | 2.250 |
| 12 | 3 | 4 | 2.864 | 1.154 | 2.387 |
|  | 6 | 6 | 2.716 | 1.108 | 2.289 |
|  | 8 | 5 | 2.719 | 1.108 | 2.286 |
|  | 8 | 8 | 2.640 | 1.091 | 2.271 |
|  | 10 | 10 | 2.640 | 1.091 | 2.271 |
| 18 | 3 | 4 | 2.870 | 1.161 | 2.394 |
|  | 6 | 6 | 2.722 | 1.116 | 2.297 |
|  | 8 | 5 | 2.725 | 1.116 | 2.294 |
|  | 8 | 8 | 2.646 | 1.099 | 2.279 |
|  | 10 | 10 | 2.645 | 1.099 | 2.279 |

**S7. Synthetic accessibility and PubChem query results**

Comprehensive details—including excited-state properties, synthetic accessibility metrics (DeepSA, SAScore, and SCScore), and PubChem query results (IUPAC names and CAS numbers)—are provided in the Excel file 'Name_DeepSA_SCS_SAS_CAS_S1o.xlsx', which is available for download on GitHub: <https://github.com/fuli-phy/SF_mols>.

**References**

[1] T. Lu, Journal of Molecular Modeling **27**, 263 (2021).

[2] J. Stoycheva, A. Tadjer, M. Garavelli, M. Spassova, A. Nenov, and J. Romanova, The Journal of Physical Chemistry Letters **11**, 1390 (2020).

[3] K. J. Fallon *et al.*, Journal of the American Chemical Society **141**, 13867 (2019).

[4] A. Stanger, The Journal of Physical Chemistry A **126**, 8049 (2022).

[5] D. Y. Zubarev and A. I. Boldyrev, Physical Chemistry Chemical Physics **10**, 5207 (2008).

[6] Z. Chen, C. S. Wannere, C. Corminboeuf, R. Puchta, and P. v. R. Schleyer, Chemical Reviews **105**, 3842 (2005).

[7] J.-i. Aihara, Journal of the American Chemical Society **128**, 2873 (2006).

[8] T. M. Krygowski, Journal of Chemical Information and Computer Sciences **33**, 70 (1993).

[9] Z. Wang, in *Chemistry*2024), pp. 1692.

[10] J. T. Blaskovits, R. Laplaza, S. Vela, and C. Corminboeuf, Advanced Materials **36**, 2305602 (2024).

[11] T. Rangel, K. Berland, S. Sharifzadeh, F. Brown-Altvater, K. Lee, P. Hyldgaard, L. Kronik, and J. B. Neaton, Physical Review B **93**, 115206 (2016).

1. Corresponding authors. *E-mail*: [zhaojj@scnu.edu.cn](mailto:zhaojj@scnu.edu.cn) (Jijun Zhao); [weiweigao@nju.edu.cn](mailto:weiweigao@nju.edu.cn) (Weiwei Gao) [↑](#footnote-ref-1)
